# Volume-wise destruction of the antiferromagnetic Mott insulating state through quantum tuning

**DOI:** 10.1038/ncomms12519

**Published:** 2016-08-17

**Authors:** Benjamin A. Frandsen, Lian Liu, Sky C. Cheung, Zurab Guguchia, Rustem Khasanov, Elvezio Morenzoni, Timothy J. S. Munsie, Alannah M. Hallas, Murray N. Wilson, Yipeng Cai, Graeme M. Luke, Bijuan Chen, Wenmin Li, Changqing Jin, Cui Ding, Shengli Guo, Fanlong Ning, Takashi U. Ito, Wataru Higemoto, Simon J. L. Billinge, Shoya Sakamoto, Atsushi Fujimori, Taito Murakami, Hiroshi Kageyama, Jose Antonio Alonso, Gabriel Kotliar, Masatoshi Imada, Yasutomo J. Uemura

**Affiliations:** 1 Department of Physics, Columbia University, New York, New York 10027, USA; 2 Laboratory for Muon Spin Spectroscopy, Paul Scherrer Institute, CH-5232 Villigen, Switzerland; 3 Department of Physics and Astronomy, McMaster University, Hamilton, Ontario, Canada L8S 4M1; 4 Canadian Institute for Advanced Research, Toronto, Ontario, Canada L8S 4M1; 5 Institute of Physics, Chinese Academy of Sciences, Beijing 100190, China; 6 Department of Physics, Zhejiang University, Hangzhou 310027, China; 7 Advanced Science Research Center, Japan Atomic Energy Agency, Tokai, Ibaraki 319-1195, Japan; 8 Department of Applied Physics and Applied Mathematics, Columbia University, New York, New York 10027, USA; 9 Condensed Matter Physics and Materials Science Department, Brookhaven National Laboratory, Upton, New York 11973, USA; 10 Department of Physics, University of Tokyo, Bunkyo-ku, Tokyo 113-0033, Japan; 11 Department of Energy and Hydrocarbon Chemistry, Graduate School of Engineering, Kyoto University, Nishikyo, Kyoto 615-8510, Japan; 12 Instituto de Ciencia de Materiales de Madrid (ICMM), CSIC, Madrid E-28049, Spain; 13 Department of Physics & Astronomy, Rutgers University, Piscataway, New Jersey 08854-8019, USA; 14 Department of Applied Physics, University of Tokyo, 7-3-1 Hongo, Tokyo 113-8656, Japan

## Abstract

RENiO_3_ (RE=rare-earth element) and V_2_O_3_ are archetypal Mott insulator systems. When tuned by chemical substitution (RENiO_3_) or pressure (V_2_O_3_), they exhibit a quantum phase transition (QPT) between an antiferromagnetic Mott insulating state and a paramagnetic metallic state. Because novel physics often appears near a Mott QPT, the details of this transition, such as whether it is first or second order, are important. Here, we demonstrate through muon spin relaxation/rotation (μSR) experiments that the QPT in RENiO_3_ and V_2_O_3_ is first order: the magnetically ordered volume fraction decreases to zero at the QPT, resulting in a broad region of intrinsic phase separation, while the ordered magnetic moment retains its full value until it is suddenly destroyed at the QPT. These findings bring to light a surprising universality of the pressure-driven Mott transition, revealing the importance of phase separation and calling for further investigation into the nature of quantum fluctuations underlying the transition.

## Abstract (web summary)

Knowing whether a quantum phase transition is first- or second-order is crucial for understanding any associated exotic phenomena, but direct experimental evidence has been scarce. Here, Frandsen *et al*. report first-order magnetic quantum phase transitions in archetypal Mott systems, providing insight into the underlying quantum fluctuations.

## Graphical abstract

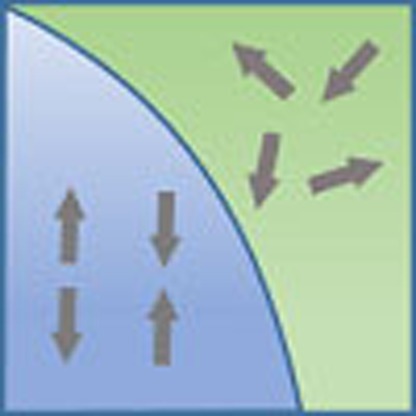

The Mott insulating phase (defined broadly as an insulating state induced by electron correlations) can often be suppressed by quantum tuning, that is, varying a nonthermal parameter such as chemical composition or pressure, resulting in a zero-temperature quantum phase transition (QPT) to a metallic state driven by quantum fluctuations^1^,^2^. Theories of exotic phenomena known to occur near the Mott QPT such as quantum criticality and high-temperature superconductivity^3^ often assume a second-order QPT, but direct experimental evidence for either first- or second-order behaviour at the magnetic QPT associated with the Mott transition has been scarce and further masked by the superconducting phase in unconventional superconductors.

The low-temperature antiferromagnetic insulating state in archetypal Mott insulators RENiO_3_ and V_2_O_3_ can be tuned systematically through variation of the rare-earth ion^4^ in RENiO_3_ and application of hydrostatic pressure^5^ in stoichiometric V_2_O_3_, making them ideal systems for studying correlation-driven metal–insulator transitions (MITs). RENiO_3_ possesses a distorted perovskite structure in which the average rare-earth ionic size can be continuously controlled by solid solution of different rare earths^4^, effectively applying a chemical pressure and producing the phase diagram shown in Fig. 1a. An abrupt thermal phase transition (solid red curve) from a high-temperature metal to a low-temperature insulator takes place at a temperature dependent on the ionic radius, with a paramagnetic–antiferromagnetic transition (blue curve) occurring simultaneously for some compounds and at lower temperature for others. A structural distortion (yellow curve) that lowers the symmetry from orthorhombic to monoclinic is also observed when cooling through the MIT^6^. As the ionic radius increases, the temperature of the thermal MIT decreases until it reaches zero at the estimated critical radius of ∼1.175 Å, resulting in a QPT from an antiferromagnetic insulator to a paramagnetic metal. Varying the rare-earth ion leaves the electron count unchanged but alters the width of the relevant electron energy bands by changing the crystal structure; hence, this constitutes a bandwidth-controlled Mott QPT^1^.

The mechanism of the MIT remains controversial. Various scenarios have been proposed, including the opening of a charge-transfer gap^4^, orbital ordering^7^ and charge ordering^6^,^8^, but experimental observations have been inconsistent with each of these scenarios^8^,^9^,^10^,^11^,^12^,^13^. Recent progress has led to a proposed site-selective Mott transition^14^,^15^ that successfully explains many of the unusual electronic properties of RENiO_3_.

In the case of V_2_O_3_, hydrostatic pressure suppresses the MIT temperature from ∼160 K at ambient pressure to 0 K at ∼2.0 GPa (Fig. 1b), again resulting in a bandwidth-controlled QPT. The MIT is likewise accompanied by structural symmetry lowering (rhombohedral to monoclinic) in the insulating state. Replacing small amounts of V with either Ti or Cr produces similar effects as pressure^16^, shown on the upper horizontal axis of Fig. 1b. Doping with Cr acts as negative pressure and leads to a first-order transition at high temperature between a paramagnetic metal and paramagnetic insulator, dividing the overall V_2_O_3_ phase diagram into three parts, similar to RENiO_3_. However, the metallic phase produced by Ti doping orders magnetically^1^ unlike the pressure-induced metal, so these tuning methods are not equivalent^17^. The MIT in V_2_O_3_ was classified early on as a Mott–Hubbard transition^5^, but experimental and theoretical developments in the 1990s and 2000s, particularly from numerical techniques like dynamical mean field theory (DMFT), modified the understanding of this system to include multiband, *S*=1 models^18^,^19^. This system remains a topic of active study.

In both materials, the QPT has received less attention than the thermal MIT, despite being vital for a full understanding of these materials and the pressure-driven Mott transition in general. The order of the QPT is significant. A second-order QPT leads to quantum criticality that may engender unusual properties and novel electronic phases. For a first-order QPT, the system would be expected to exhibit more typically first-order behaviour such as phase coexistence and abrupt changes in the ground state, not necessarily manifesting quantum criticality in the same way. Early numerical and analytical studies^20^,^21^,^22^,^23^,^24^, scaling analysis^25^ and DMFT studies^26^ suggested first-order behaviour at the Mott QPT, but conclusive experiments on RENiO_3_ and V_2_O_3_ have been lacking. Among the few experiments related to the QPT in these materials are a transport study of V_2_O_3_ under pressure^27^ revealing a gradual reduction of the low-temperature resistance with higher pressure, a neutron study^28^ suggesting quantum critical spin fluctuations in heavily Ti-doped V_2_O_3_, and scanning tunnelling spectroscopy measurements of thin-film NdNiO_3_ and LaNiO_3_ that has been interpreted as evidence for a quantum critical point^29^. However, much remains unknown about the QPT in both of these systems, in part because volume-integrating probes like transport and neutron scattering are typically unable to distinguish between second-order and first-order transitions with phase separation.

We have utilized muon spin relaxation/rotation (μSR) to study the QPT in RENiO_3_ and V_2_O_3_. A highly sensitive probe of local magnetism, μSR can measure the local order parameter and magnetically ordered volume fraction independently, and is therefore ideally suited to determine first- or second-order behaviour at a QPT. This makes μSR highly complementary to probes of long-range magnetic order such as neutron diffraction. The results presented here unambiguously demonstrate that the QPT from antiferromagnetic insulator to paramagnetic metal in RENiO_3_ and V_2_O_3_ is first order. On approaching the QPT from the ordered side, the magnetically ordered volume fraction decreases steadily until it reaches zero at the QPT, resulting in a broad region of intrinsic phase separation, while the ordered magnetic moment retains its full value across the phase diagram until it is suddenly destroyed at the QPT. These findings bring to light a surprising universality of the pressure-driven Mott transition in three spatial dimensions, revealing the importance of phase separation in a significant region of parameter space near the QPT and calling for further investigation into the role of inelastic soft modes and the nature of dynamic spin and charge fluctuations underlying the transition.

## Results

### Composition-tuned QPT in RENiO_3_

**Figure 1:**
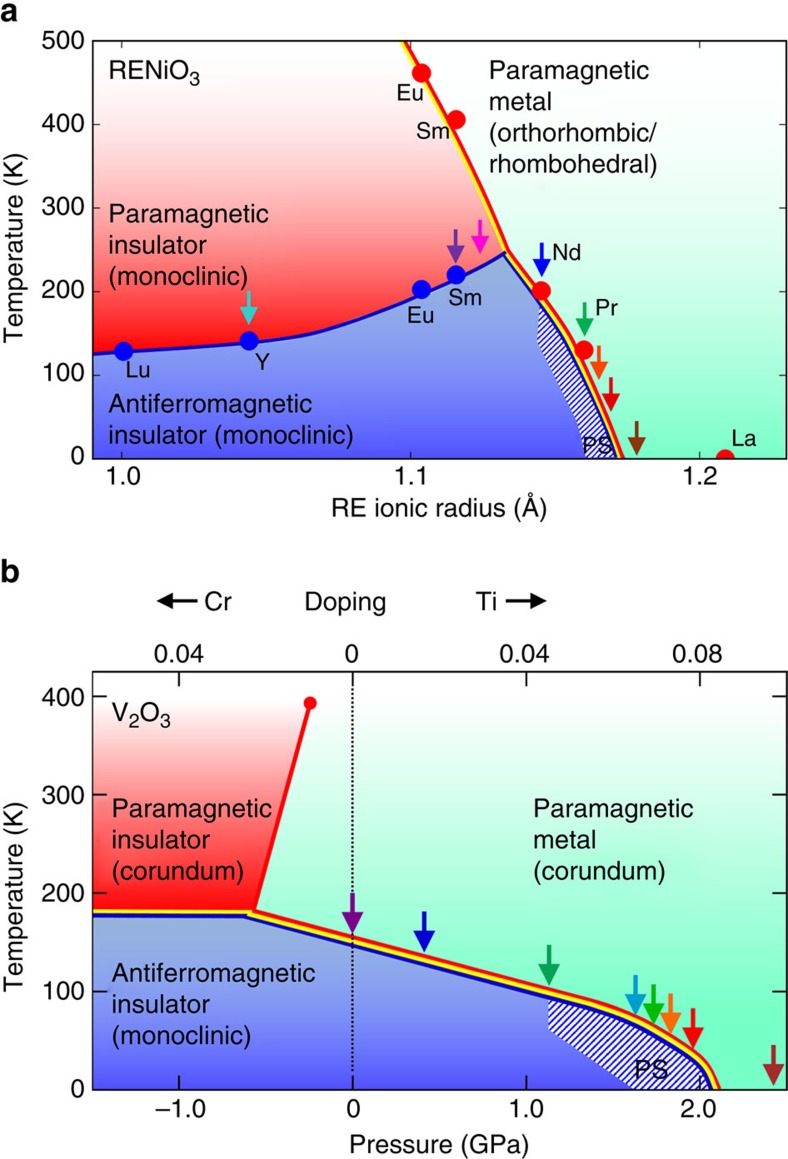
**Phase diagrams for canonical Mott systems RENiO**_**3**_ **and V**_**2**_**O**_**3**_. (**a**) RENiO_3_ phase diagram, with temperature along the vertical axis and rare-earth ionic radius along the horizontal axis. The red curve indicates a metal–insulator transition on cooling, blue a paramagnet–antiferromagnetic transition, and yellow a structural transition. The coloured circles represent phase boundaries for the stoichiometric compounds determined by previous studies. Coloured arrows indicate compositions studied in the current work. The quantum phase transition (QPT) occurs at a radius of ∼1.175 Å. PS, phase separated. After ref. 4. (**b**) V_2_O_3_ phase diagram, with temperature along the vertical axis and hydrostatic pressure along the horizontal axis. The QPT occurs at a pressure of ∼2.0 GPa. Doping with Ti and Cr is shown on the upper horizontal axis for comparison. All coloured curves and symbols are the same as in **a**. After ref. 16.

The compositions of RENiO_3_ studied in the present work include are indicated by the coloured arrows in Fig. 1a: RE=Y (cyan), Sm (purple), Sm_0.75_Nd_0.25_ (pink), Nd (blue), Pr (green), Nd_0.7_La_0.3_ (orange), Nd_0.6_La_0.4_ (red) and Nd_0.5_La_0.5_ (brown). μSR experiments in zero externally applied magnetic field (ZF) and a weak transverse field (wTF) applied perpendicular to the initial muon spin direction were performed for each sample on a temperature grid spanning the thermal magnetic phase transition, providing a detailed picture of the phase diagram near the QPT.

**Figure 2:**
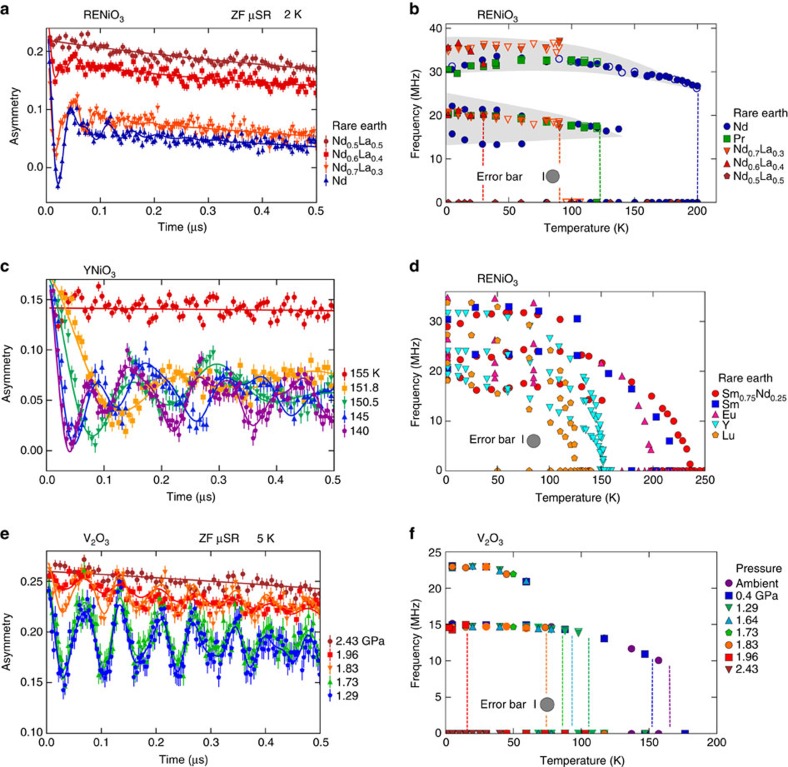
**Zero-field (ZF) muon spin relaxation (μSR) experiments on RENiO**_**3**_ **and V**_**2**_**O**_**3**_. (**a**) ZF time spectra taken at 2 K for four compounds of RENiO_3_ near the quantum phase transition. The coloured dots represent the data, the solid curves the fits. The three magnetically ordered compounds show nearly identical oscillation frequencies (hence identical moment sizes) but very different oscillation amplitudes (hence different ordered volume fractions). (**b**) Temperature dependence of the oscillation frequencies for RENiO_3_ compounds with first-order thermal phase transitions. Filled (open) circles represent data taken in a cooling (warming) sequence. All magnetically ordered compounds have two or three frequencies lying along two common bands (shaded grey regions), indicating that the ordered moment size does not change along the horizontal axis of the phase diagram. The large grey circle with the neighbouring vertical bar indicates the average estimated standard deviation (ESD) of the refined frequency produced from the least-squares minimization compared to the symbol size. The coloured dashed lines are guides to the eye showing the approximate transition temperature for each composition. (**c**) ZF spectra for YNiO_3_ taken at various temperatures through the thermal phase transition. (**d**) Temperature dependence of the oscillation frequencies for RENiO_3_ compounds with second-order-like thermal phase transitions, revealing the continuous development of the ordered moment size. (**e**,**f**) Plots for pressure-tuned V_2_O_3_ corresponding to (**a**,**b**).

Representative ZF time spectra taken at 2 K on four compositions of RENiO_3_ near the QPT are displayed in Fig. 2a. The spectrum for Nd_0.5_La_0.5_NiO_3_ exhibits only slow relaxation with no oscillations, indicating an absence of magnetic order, as expected for this composition. In contrast, damped but coherent oscillations are seen for Nd_0.6_La_0.4_NiO_3_, placing this composition just to the left of the QPT. Nd_0.7_La_0.3_NiO_3_ and NdNiO_3_ likewise show oscillations indicative of magnetic ordering.

For a given material, the ZF oscillation frequency and amplitude are directly proportional to the ordered moment size and the magnetically ordered volume fraction of the sample, respectively. From Fig. 2a, one clearly observes that the three ordered compounds have approximately the same oscillation frequency (hence moment size), but different oscillation amplitudes (hence OVFs). To better illustrate the frequency behaviour, we plot in Fig. 2b the temperature dependence of the oscillation frequency for the compounds closest to the QPT. These frequencies were extracted from refinements described in the Methods section. The most striking feature of these results is that the two or three oscillation frequencies observed in each of the ordered compounds all lie along the same two frequency bands, illustrated by the shaded grey regions in Fig. 2b. This indicates that the saturated moment size is not changed by compositional tuning towards the QPT; rather, the full moment is destroyed abruptly and discontinuously at the QPT.

The compounds represented in Fig. 2b lie in the region of the phase diagram where the magnetic transition occurs simultaneously with the electronic and structural transitions. The temperature dependence of the frequency shows a discontinuous onset at the ordering temperature for these compounds, corresponding to a first-order thermal phase transition. We also measured several compounds located further away from the QPT where the magnetic transition is split from the electronic and structural transitions. Representative ZF spectra for YNiO_3_ taken at several temperatures spanning the thermal phase transition are shown in Fig. 2c, revealing a gradual increase in the precession frequency as the temperature is lowered. This is further illustrated in Fig. 2d for RE=Lu, Y, Eu, Sm, and Sm_0.75_Nd_0.25_, with the continuous onset of the oscillation frequency suggesting second-order-like behaviour of the thermal phase transition. This is consistent with previous studies indicating a second-order magnetic transition for compounds with split electronic/structural and magnetic transitions, and first-order behaviour when the transitions occur simultaneously^30^.

**Figure 3:**
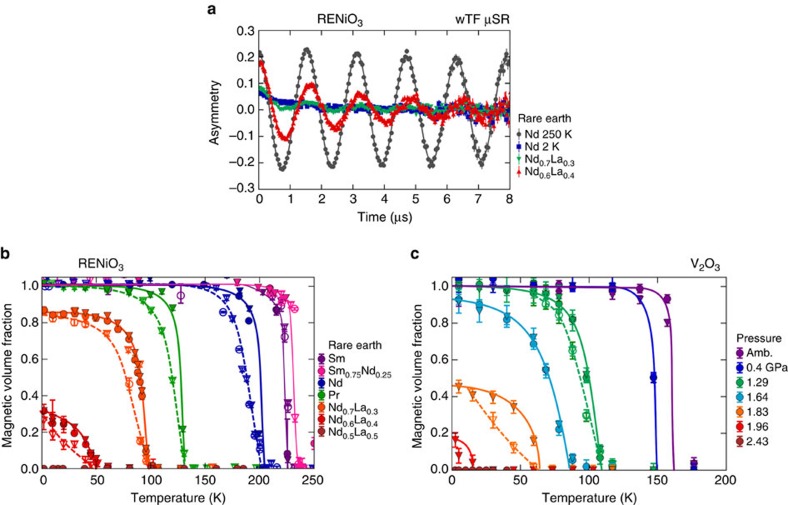
**Weak transverse field (wTF) muon spin rotation (μSR) experiments and magnetic volume fraction of RENiO**_**3**_ **and V**_**2**_**O**_**3**_. (**a**) wTF time spectra for three compounds of RENiO_3_ near the quantum phase transition (QPT) at 2 K, with one spectrum measured at higher temperature (250 K) shown for comparison. The coloured dots represent the data, the solid curves the fits described in the text. (**b**) Temperature dependence of the magnetic volume fraction in RENiO_3_ derived from the fits. Circles (triangles) represent wTF (ZF) measurements, and filled (open) symbols represent warming (cooling) sequences. Solid and dashed curves are guides to the eye, with solid corresponding to cooling and dashed to warming. The compounds near the QPT have a significantly reduced ordered volume fraction at low temperature, indicating phase separation between magnetic and paramagnetic regions of the sample. Error bars were obtained by propagating the estimated standard deviations (ESDs) of the refined asymmetry values produced from the least-squares minimization. (**c**) Magnetic volume fraction of V_2_O_3_ under different hydrostatic pressures. The symbols are the same as in **b**. As with RENiO_3_, the ordered volume fraction is strongly reduced near the QPT.

The reduced ZF oscillation amplitudes observed in Fig. 2a for Nd_0.6_La_0.4_NiO_3_ and Nd_0.7_La_0.3_NiO_3_ suggest that even at 2 K, these compounds are not magnetically ordered throughout the full volume fraction, rather there are some paramagnetic regions remaining. To verify this, we performed wTF experiments at several temperatures for each compound. The results are summarized in Fig. 3a,b. Figure 3a displays the time spectra for Nd_0.6_La_0.4_NiO_3_, Nd_0.7_La_0.3_NiO_3_, and NdNiO_3_ at 2 K and NdNiO_3_ at 250 K. In wTF, the amplitude of the low-frequency oscillations is proportional to the non-magnetically ordered volume fraction; thus, a spectrum with no oscillation (such as NdNiO_3_ at 2 K) corresponds to a fully ordered sample, while a spectrum with oscillation in the full asymmetry (such as NdNiO_3_ at 250 K) indicates a completely disordered sample. The spectra for Nd_0.7_La_0.3_NiO_3_ and Nd_0.6_La_0.4_NiO_3_ show intermediate oscillation amplitudes, indicating that even at 2 K, these materials are only partially ordered. These compounds therefore exhibit intrinsic paramagnetic and antiferromagnetic phase separation.

The temperature dependence of the magnetic volume fraction extracted from the wTF data for several compounds spanning the phase diagram is displayed by the circles in Fig. 3b, along with the results determined from the ZF experiments shown as triangles. Nd_0.7_La_0.3_NiO_3_ and Nd_0.6_La_0.4_NiO_3_, the compounds that are closest to the QPT, show a significantly reduced ordered volume fraction, verifying that their ground state consists of phase-separated antiferromagnetic and paramagnetic regions. PrNiO_3_ and NdNiO_3_ also show phase separation over a broad temperature interval of about 50 K below the onset ordering temperature, although both are fully ordered at lower temperatures. The region of the phase diagram exhibiting phase-separated behaviour is indicated by the shaded area marked PS in Fig. 1. This magnetic phase separation may also be accompanied by phase separation between the high-temperature and low-temperature structural and electronic phases, but that is beyond the scope of this study. It is interesting to note that for the compounds exhibiting phase separation, the transition is stretched over a rather large temperature range. The hysteretic differences between measurements taken in cooling and warming sequences (represented by open and filled symbols, respectively) confirms the first-order nature of thermal phase transition for NdNiO_3_, PrNiO_3_, Nd_0.7_La_0.3_NiO_3_ and Nd_0.6_La_0.4_NiO_3_.

Importantly, the reduced magnetic volume fraction in Nd_0.7_La_0.3_NiO_3_ and Nd_0.6_La_0.4_NiO_3_ cannot be attributed merely to an extrinsic effect of doping, since the doped compound Sm_0.75_Nd_0.25_NiO_3_ shows a rapid magnetic transition in the full volume fraction. Furthermore, neutron diffraction of the Nd_0.6_La_0.4_NiO_3_ sample at 70 K indicates that it is single-phase (Supplementary Fig. 2), excluding poor sample quality as a cause of this behaviour. Therefore, we attribute the reduced volume fraction to proximity to the QPT. X-ray diffraction measurements on NdNiO_3_ also confirm that the expected structural response at the MIT occurs with hysteresis over a similar temperature range as that of the magnetic hysteresis, verifying the correlation between the magnetic and structural phases (Supplementary Fig. 1).

### Pressure-tuned QPT in V_2_O_3_

To investigate whether the volume-wise destruction of the AF Mott phase at the QPT is a feature unique to RENiO_3_ or is perhaps more generic, we performed corresponding ZF and wTF experiments on pressure-tuned V_2_O_3_. Hydrostatic pressure has the advantage of being a very clean tuning parameter, avoiding any possible complications of structural or chemical inhomogeneity often associated with chemical doping. The pressures at which we measured V_2_O_3_ are indicated by coloured arrows in Fig. 1b: ambient pressure, 0.4, 1.29, 1.64, 1.73, 1.83, 1.96 and 2.43 GPa.

The key results for V_2_O_3_ are identical to those for RENiO_3_: the AF state is destroyed by reduction of the ordered volume fraction while the moment size remains constant, with a significant region of ground-state phase separation near the QPT. The ZF μSR results for V_2_O_3_ are summarized in Fig. 2e,f. From the ZF spectra displayed in Fig. 2e, it is clear that the oscillation frequencies at various pressures are virtually identical, while the oscillation amplitude shows a significant decrease as the critical pressure of ∼2.0 GPa is approached. As seen in Fig. 2f, all ZF oscillation frequencies lie along the same two frequency bands, demonstrating that pressure does not affect the ordered moment size. The lower-frequency band is in good agreement with an early μSR experiment^31^ on V_2_O_3_. The ordered volume fraction obtained by both the ZF and wTF experiments is displayed in Fig. 3c, where we observe a gradual reduction in the low-temperature magnetic volume fraction beginning with the 1.64 GPa measurement. At 1.96 GPa, only about 15% of the volume is magnetically ordered, and at 2.43 GPa, the magnetism is completely absent. The phase separation between AF and paramagnetic regions is reminiscent of that observed in the charge sector by spatially resolved infrared spectroscopy in pure and Cr-doped V_2_O_3_ (ref. 32). The curious stretching of the magnetic transition observed in RENiO_3_ is also seen in V_2_O_3_ for larger applied pressures, in agreement with an earlier high-pressure NMR study^33^. This stretched transition is also somewhat similar to that observed in V_2_O_3_ nanocrystals^34^, although surface area effects are likely the cause in that case. These results demonstrate that the AF phases in RENiO_3_ and V_2_O_3_ have identical behaviour in all key aspects near the QPT.

## Discussion

The two main results of these experiments are (1) that the saturated magnetic moment size is constant across the ordered region of the phase diagram before being abruptly destroyed at the QPT; and (2) that the magnetic volume fraction is heavily reduced near the QPT. We observed identical behaviour for RENiO_3_ and pressure-tuned V_2_O_3_, both canonical bandwidth-controlled Mott insulators. These results unambiguously demonstrate that the QPT in these materials proceeds in a distinctly first-order fashion. The striking similarity between both of these canonical systems, along with experimental indications of structural phase separation in the archetypal Mott system NiS_2_ tuned by pressure^35^, indicates that this is a generic feature of the pressure-driven Mott QPT, supporting previous theoretical work^23^,^24^,^25^,^26^,^36^. Further theoretical considerations regarding the surprisingly large region of parameter space exhibiting phase coexistence and additional details of this first-order QPT are provided in the Supplementary Note 1.

We note that similar phase separation between paramagnetic and magnetically ordered regions was observed by μSR^37^ and nuclear magnetic resonance^38^ in the destruction of magnetic order in the itinerant helimagnet MnSi through pressure tuning, and by μSR^37^ in the metallic ferromagnet (Sr,Ca)RuO_3_ through (Sr,Ca) chemical substitution. Signatures of first-order magnetic quantum evolution have also been observed in heavy fermion systems^37^ and many unconventional superconductors^37^,^39^, accompanied by an inelastic resonance mode in the superconducting state^39^,^40^. The magnetic resonance mode can be viewed as a soft mode towards the parent/competing magnetic state appearing due to the closeness of free energies of the magnetically ordered and superconducting states across the first-order transition^39^. Such a soft mode may also exist in the paramagnetic metallic state of non-superconducting Mott transition systems near the QPT as a signature of the imminent AF-insulating electronic structure appearing in the dynamic and inelastic spin/charge correlations. The pseudogap observed in tunnelling experiments^29^ on paramagnetic metallic LaNiO_3_ may be viewed as a charge soft mode in a magnetically disordered metallic state adjacent to the Mott insulator NdNiO_3_. Further studies of antiferromagnetic Mott insulators and other quantum magnetic systems will be useful to elucidate generic and system-specific roles of first-order behaviour in quantum phase evolution.

## Methods

### Specimen preparation

The RENiO_3_ perovskites were prepared in polycrystalline form as follows: analytical grade R_2_O_3_ (R=La,Pr,Nd,Sm) and Ni(NO_3_)_2_·6H_2_O were solved in citric acid with some droplets of nitric acid; the mixture of citrate and nitrate solutions was slowly evaporated, leading to organic resins, which were dried and decomposed by slowly heating up to 800 °C in air, for 12 h. This treatment gave rise to highly reactive precursor materials, amorphous to X-ray diffraction. The precursor powders were treated at 900 °C under 200 bar of O_2_ pressure for 12 h in a Morris Research furnace. Then the samples were slowly cooled down (2 °C min^−1^) to room temperature. Finally, the samples were pelletized and re-treated at 900 °C under O_2_ pressure for 12 h to give 6 mm disks suitable for the μSR experiments. Another set of (Nd,La)NiO_3_ samples for additional X-ray measurement was prepared from a stoichiometric mixture of RE_2_O_3_(99.9%), NiO(99.9%) and KClO_4_ (99, 100% overweight). The mixtures were placed in a Au capsule and treated at 6 GPa in a cubic-anvil-type high-pressure apparatus at 1,100 °C for 30 min before being quenched to room temperature and subsequently releasing the pressure. After removing the capsule, the sample was crushed and washed in distilled water to dissolve the KCl and obtain the pure RENiO_3_ sample. The resulting RENiO_3_ purified powder was heated in an evacuated oven at 150 °C for 30 h to evaporate any remaining water. In all samples prepared, the presence of any possible impurity phase can be limited to well below 2% based on structural characterization from X-ray and neutron diffraction analysis. Additional discussion of the sample quality is provided in the Supplementary Note 2 and Supplementary Fig. 1. The large polycrystalline specimen of V_2_O_3_ was synthesized via reduction of high-purity V_2_O_5_ in 5% H_2_/Ar gas at 900 °C for 48 h, followed by cooling at 100 °C/h.

### μSR experiments

The μSR technique exploits the asymmetric decay of muons into positrons to act as a highly sensitive probe of local magnetism. The μSR experiments on RENiO_3_ were conducted at the Centre for Molecular and Materials Science at TRIUMF in Vancouver, Canada using the LAMPF spectrometer, and the pressure-dependent μSR experiments on V_2_O_3_ were conducted at the Paul Scherrer Institute in Villigen, Switzerland using the GPD instrument. In both cases, a gas-flow cryostat was used, providing access to temperatures from 2 K to room temperature. Hydrostatic pressure was applied to V_2_O_3_ by immersing the sample in Daphne oil in a double-walled cylindrical piston cell made of MP35N material. The pressure was calibrated by observing the change in the superconducting transition temperature of a small indium plate placed in the oil with the sample. The uncertainty in the measured pressure was less than 0.05 GPa.

The μSR spectra were analysed using the least-squares minimization routines in the MusrFit software package^41^. The wTF asymmetry spectra were modelled by the function


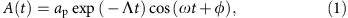


where *t* is time after muon implantation, *A*(*t*) is the time-dependent asymmetry, *a*_p_ is the amplitude of the oscillating component (related to the paramagnetic volume fraction), Λ is an exponential damping rate due to paramagnetic spin fluctuations and/or nuclear dipolar moments, ω is the Larmor precession frequency set by the strength of the transverse magnetic field, and *φ* is a phase offset. In the case of V_2_O_3_, a second slowly-damped oscillating component was included to account for muons stopping in the pressure cell. The zero for *A*(*t*) was allowed to vary for each temperature to deal with the asymmetry baseline shift known to occur for magnetically ordered samples, as has been done elsewhere^42^. From these refinements, the magnetically ordered volume fraction at each temperature *T* was estimated as 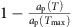, where *a*_p_(*T*_max_) is the amplitude in the paramagnetic phase at high temperature. We performed a second set of refinements of the wTF data using a two-component model for the asymmetry, an oscillating component with asymmetry *a*_p_ representing the paramagnetic fraction and a non-oscillating exponentially decaying component with asymmetry *a*_o_ representing the 1/3 component of the magnetically ordered fraction. The total initial asymmetry *a*_tot_=*a*_p_+*a*_o_ and the asymmetry baseline fixed to the values determined from a high-temperature measurement well above the magnetic ordering temperature. Both methods of analysis of the wTF data yielded essentially identical results for the magnetic volume fraction. The results from the first method of refinement are shown in Fig. 3.

The ZF asymmetry spectra were modelled by the function


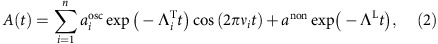


where the 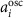 are the amplitudes of the precessing components, the 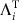 are the transverse damping rates due to a distribution of field strengths at the muon site(s), the *ν*_*i*_ are the precession frequencies, *a*^non^ is the asymmetry of the non-oscillating component due to a combination of paramagnetic regions and the 1/3 non-oscillating component arising from the orientationally averaged magnetic regions of the sample, and Λ^L^ is the longitudinal relaxation rate (also known as 1/T_1_ when it applies to the 1/3 component from magnetically ordered regions). In all cases except for RE=Nd, there are two oscillating components at the lowest temperatures, with the lower-frequency component having roughly double the amplitude of the higher-frequency component. This may be understood as arising from two magnetically inequivalent muon stopping sites. For RE=Nd, a third frequency can be resolved at the lowest temperatures, perhaps due to ordering of the Nd moments. For the V_2_O_3_ experiments, a damped Kubo-Toyabe component with fixed amplitude and field width was also included to capture the contribution from muons stopping in the pressure cell. The total initial asymmetry was ∼0.258, with 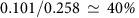 of the signal arising from muons stopping in the sample. The highest temperature at which spontaneous oscillations of the ZF asymmetry are observed was used to define the magnetic phase boundary.

The precession frequencies were determined from refinements performed over the first μs of the asymmetry time spectra. Additional refinements over a larger time window (0–8 μs) and a larger time-channel binning size were performed to more accurately determine *a*^non^ and Λ^L^. For a magnetically ordered sample with domains distributed isotropically overall solid angles, 2/3 of the asymmetry will exhibit oscillations and 1/3 will not. This is because magnetic field components along the Cartesian direction defined by the initial muon spin do not contribute to the precession of the muon spin, while the other two Cartesian directions do. In RENiO_3_ and V_2_O_3_, as in many other magnetic materials, the 2/3 oscillating component is strongly damped, and is manifest only as missing asymmetry when the spectrum is viewed over a large time window with relatively large time-channel binning. The total asymmetry arising from magnetically ordered regions of the sample can therefore be estimated as 3/2 multiplied by the missing asymmetry, with the magnetic volume fraction then given by 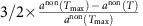. For RENiO_3_, the magnetic volume fraction determined from ZF data in this way agreed quite well with the volume fraction determined by the wTF experiments. For V_2_O_3_, this resulted in a slight overestimation of the magnetic volume fraction. Therefore, we multiplied by 4/3 instead of 3/2 (corresponding to a 1/4 non-oscillating component rather than 1/3), which then gave close agreement with the wTF results. Such a deviation from the ideal 2/3 versus 1/3 division of the asymmetry is not uncommon and simply reflects an imperfect orientational average of the magnetic domains.

### X-ray and neutron diffraction measurements

X-ray diffraction measurements of NdNiO_3_ were performed at NSLS-II at Brookhaven National Laboratory on the XPD instrument^43^,^44^. The temperature was controlled with a gas cryostream. Rietveld refinements^45^ of the diffraction patterns were performed using the Full Prof software suite^46^, and pair distribution functions (PDFs) were generated and modelled using the xPDFsuite programme^47^,^48^. The orthorhombic Pbnm structure was used to model the data at all temperatures. This was sufficient to verify the presence of the expected structural change over the same temperature region as the magnetic transition, even though the true low-temperature structure has monoclinic symmetry. Supplementary Fig. 2 displays the temperature dependence of the unit cell volume in panel (a) and the isotropic atomic displacement parameter for Ni in panel (b), both obtained from PDF modelling. In both cases, a deviation from the high-temperature trend is observed at 200 K, with substantial differences between the warming and cooling sequences over a similar temperature range as the magnetic volume fraction hysteresis observed by μSR. The results of the Rietveld refinements were very similar. These observations are consistent with previous structural studies of NdNiO_3_.

Time-of-flight neutron diffraction measurements of Nd_0.6_La_0.4_NiO_3_ were performed at the Spallation Neutron Source of Oak Ridge National Laboratory on the NOMAD instrument. Rietveld refinements were performed using GSAS^49^ on the EXPgui platform^50^. Supplementary Fig. 3 displays the results of refining the Pbnm model against the measured diffraction pattern at 70 K, which is above the MIT in this compound. The measured pattern is shown by blue crosses, the calculated pattern in red, and the difference curve in green. The fit is of good quality, ruling out the presence of any significant impurity phase in this compound.

### Data availability

All relevant data are available from the authors upon request.

## Additional information

**How to cite this article:** Frandsen, B. A. *et al*. Volume-wise destruction of the antiferromagnetic Mott insulating state through quantum tuning. *Nat. Commun.* 7:12519 doi: 10.1038/ncomms12519 (2016).

## Supplementary Material

Supplementary Information Supplementary Figures 1-3, Supplementary Notes 1-2 and Supplementary References
