# Supplementary material for: Volume-wise destruction of the antiferromagnetic Mott insulating state through quantum tuning
Source: Nat Commun. 2016 Aug 17;7:12519. doi: 10.1038/ncomms12519 (PMC4992057; doi:10.1038/ncomms12519)
Supplement: Supplementary Information — Supplementary Figures 1-3, Supplementary Notes 1-2 and Supplementary References [file ncomms12519-s1.pdf]

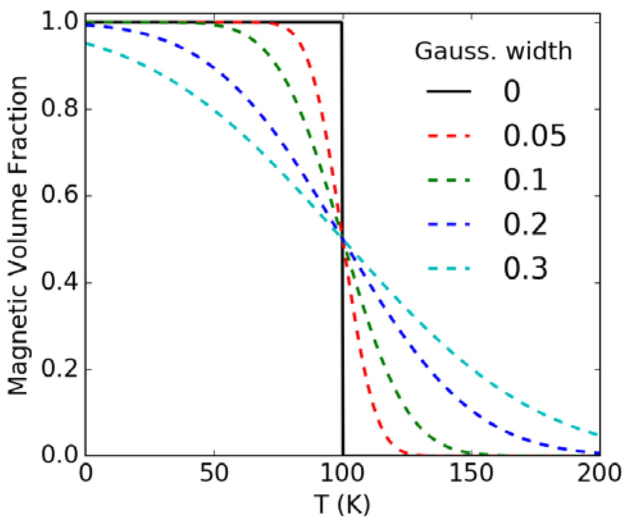

Supplementary Figure 1. Simulated temperature dependence of the magnetically ordered volume fraction for compositional inhomogeneity. A Gaussian distribution of the concentration of rare-earth ions around the nominal composition was considered, with the Gaussian widths indicated in the figure.

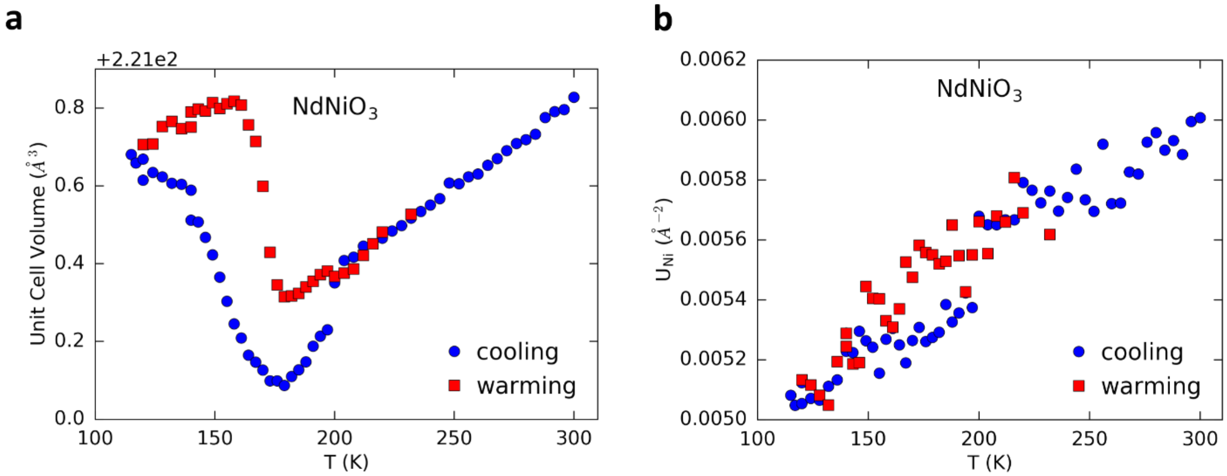

Supplementary Figure 2. Selected results of x-ray pair distribution function measurements of $NdNiO_3$. (a) Unit cell volume obtained from refined lattice parameters. A clear response is observed at 200 K with significant hysteresis over a large temperature region, consistent with the μSR results. (b) Isotropic atomic displacement parameter *U* for Ni, again showing a hysteretic response below 200 K.

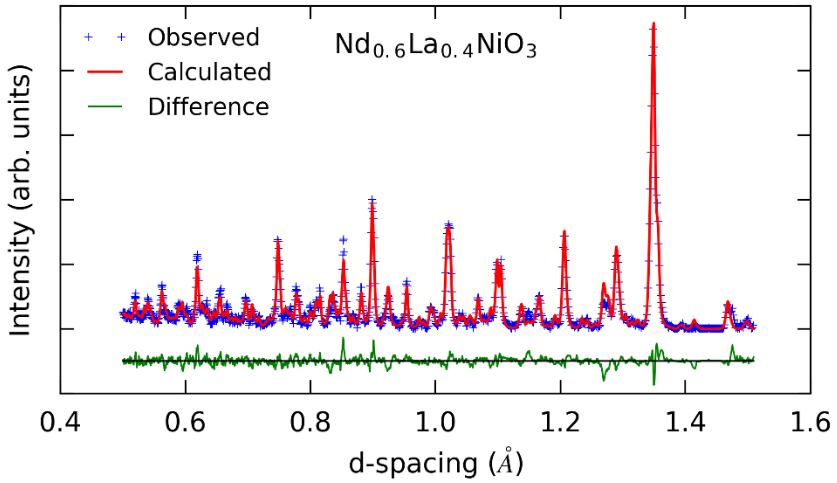

Supplementary Figure 3. Neutron diffraction measurement of $Nd_{0.6}La_{0.4}NiO_3$ at 70 K. The blue crosses represent the measured intensity, the red curve the calculated pattern, and the green curve is the difference.

Supplementary Note 1: Theoretical considerations. In an ideal system, the pressure-driven MIT should occur at one specific pressure for any given temperature. However, the disorder resulting from the use of chemical pressure and the finite time scales involved in the experimentation necessarily introduce microscopic inhomogeneities and non-equilibrium phenomena, which are manifest in a finite interval of pressures where the two phases coexist. Additionally, the assumption of constant pressure may be unrealistic at the microscopic level due to local variations in unit cell structure and/or size, causing microscopic strains near domain boundaries, for example. This further leads to inhomogeneous nucleation during the phase transition. Recent theoretical work of Yee and Balents[1] also showed phase separation between antiferromagnetic Mott insulating and paramagnetic metallic states via charge doping, but this may not be directly applicable to the present work due to differences in the tuning methods and the underlying physical assumptions.

The universality brought to light in this paper suggests that while magnetism and structural changes accompany the Mott transition, these effects are not likely to be essential to its existence, since they are present with various strengths in different compounds. Earlier theoretical results[2-5] and the experimental results presented in this work suggest that the strength of the first-order transition can be varied, and decreased with increasing quantum fluctuations to approach an interesting marginal point which separates the first order regime from a regime where the Mott transition is continuous. The marginal point may offer novel physics associated with the quantum criticality distinct from the conventional one driven by the spontaneous symmetry breaking. The pressure-driven metal-insulator coexistence region revealed in the present paper provides us with the possibility of unique quantum fluctuations reminiscent of, but distinct from, the inhomogeneity extensively studied in the filling controlled case such as the high-T_c cuprates and the colossal magnetoresistive perovskite manganites. The generality of the first-order nature of the Mott transition across different series of materials calls for the determination of its strength. Detailed studies of transport coefficients[6], optical conductivities, and sound velocity measurements coupled with system specific calculations which go beyond model studies will be needed to answer this question.

Supplementary Note 2: Assessing sample quality. One potential concern with the results reported in this work is that the solid solution $RENiO_3$ samples with Nd and La may not have a uniform composition throughout the entire sample. Such compositional variation would be expected to affect the temperature dependence of the magnetically ordered volume fraction. If the local chemical composition within the sample were to vary, for example as a Gaussian distribution of some width centered on some average composition, then the local magnetic ordering temperature would depend on the local La content according to the established phase diagram. This means that for any given average composition of the sample, La-poor regions would order at higher temperature, La-rich regions at lower temperature, and if close enough to the QPT, regions with high enough local La content would possibly not order at all. If these latter regions existed in the sample, their presence could explain why the magnetic volume fraction is less than unity near the QPT. To rule this out, we simulated the temperature dependence of the magnetically ordered volume fraction in such a situation for several different Gaussian widths of the chemical composition, which we show in Supplementary Figure 3.

The most striking feature of these simulations is that the temperature dependence of the ordered fraction becomes rounded above and below the average ordering temperature ($T_{N\text{-avg}}$; 100~K in these examples), with an inflection point occurring at $T_{N\text{-avg}}$. The current μSR data for $RENiO_3$ show no such high-temperature tails of the ordered volume fraction; rather, they show a rather sharp onset of the ordered volume fraction, which then gradually flattens out and ends up at a maximum value less than unity for the compositions with 30% and 40% La content. The lack of this distinctive rounding above the average ordering temperature in our data is strong evidence against the possibility of significant compositional variation within our samples.

It is also important to establish that the polycrystalline nature of the samples studied in this work does not affect the interpretation of the reduced magnetic volume fraction near the QPT, for instance due to the increased ratio of the surface area to volume. A few considerations show that this is not the case. The polycrystalline samples used in this study have a relatively large grain size (~1-5 μm), corresponding to well-crystallized samples. Furthermore, the samples were annealed at relatively high temperature (~900 K), where crystalline growth is notable. Therefore, these samples are far away from the nanocrystalline regime where surface effects become important. In addition, the sharp onset of magnetic order in V_2O_3 at ambient pressure and in the $RENiO_3$ samples well away from the QPT is in stark contrast to the very gradual development of magnetic order as the temperature is lowered for nanocrystalline V_2O_3, studied by μSR some years ago[7].

Supplementary References

[1] Yee, C.-H. & Balents, L. Phase separation in doped Mott insulators. *Phys. Rev. X* **5**, 021007 (2015).
[2] Watanabe, S. & Imada, M. Precise determination of phase diagram for two-dimensional Hubbard model with filling- and bandwidth-control Mott transitions: Grand-canonical path-integral renormalization group approach. *Journal of the Physical Society of Japan* **73**, 1251-1266 (2004).
[3] Misawa, T. & Imada, M. Quantum criticality around metal-insulator transitions of strongly correlated electron systems. *Phys. Rev. B* **75**, 115121 (2007).

[4] Imada, M. Universality classes of metal-insulator transitions in strongly correlated electron systems and mechanism of high-temperature superconductivity. *Phys. Rev. B* **72**, 075113 (2005).
[5] Chitra, R. & Kotliar, G. Dynamical mean field theory of the antiferromagnetic metal to antiferromagnetic insulator transition. *Phys. Rev. Lett.* **83**, 2386-2389 (1999).
[6] Husmann, A. *et al.* Dynamical signature of the Mott-Hubbard transition in $Ni(S,Se)_2$. *Science* **274**, 1874-1876 (1996).
[7] Blagojevic, V. A. *et al.* Magnetic phase transition in V_2O_3 nanocrystals. *Phys. Rev. B* **82**, 094453 (2010).
